# Supplementary material for: Vascular Effects, Potential Pathways and Mediators of Fetal Exposure to Alcohol and Cigarette Smoking during Pregnancy: A Narrative Review
Source: Int J Environ Res Public Health. 2023 Jul 19;20(14):6398. doi: 10.3390/ijerph20146398 (PMC10378932; doi:10.3390/ijerph20146398)
Supplement: Supplementary file 1 [file ijerph-20-06398-s001.zip › ijerph-2358214-supplementary.pdf]

Supplementary File S1:

Table S1: SCOPUS search strategy

Search #	Search terms TITLE-ABS-KEY
#1	TITLE-ABS-KEY ((“maternal smoking”) OR (“tobacco smoke expos*”) OR (“smoking during pregnancy”) OR (“prenatal smok*”) OR (‘smoking pregnant) OR (“cigarette smoking”) OR (“maternal smok*”)
#2	TITLE-ABS-KEY ((“alcohol exposure”) OR (“maternal alcohol exposure”) OR (“maternal alcohol consumption”) OR (“fetal alcohol*”) OR (“alcohol exposure in utero”) OR (“Ethanol”) OR (“ethanol exposure”))
#3	TITLE-ABS-KEY ((“intima media thickness”) OR (“carotid intima media thickness”) OR (“cIMT“) OR (“aortic intima media thickness“)OR (“aIMT“) OR (“vascular dysfunction“) OR (“atherosclero*“) OR (“vascular stiffness“) OR (“arterial stiffness“) OR (“pulse wave velocity“) OR (“PWV“))
#4	TITLE-ABS-KEY ((“children”) OR (“childhood”) OR (“infants”) OR (“adolescents”) OR (“adolescence”) OR (“child”) OR (“neonates”) OR (“neonatal”) OR (“newborns”) OR (“Newborn infants”) OR (“offspring”) OR (“animals”))
#5	TITLE-ABS-KEY ((1 AND 3 AND 4) OR (2 AND 3 AND 4) OR (1 AND 2 AND 3 AND 4))

Table S2: Web of Science search strategy

Search #	**Search terms**
#1	(TS= ((”maternal smoking”) OR (“tobacco smoke expos*”) OR (“smoking during pregnancy”) OR (“prenatal smok*”) OR (”smoke pregnant”) OR (”smoking pregnant”) OR (“smoking during pregnancy”) OR (”cigarette smoking”) OR (“maternal smok*”)))
#2	(TS= ((”alcohol exposure”) OR (”maternal alcohol exposure”) OR (”maternal alcohol consumption”) OR (”fetal alcohol”) OR (”foetal alcohol”) OR (”fetal alcohol exposure”) OR (”foetal exposure”) OR (”alcohol exposure in utero”) OR (”ethanol”) OR (”ethanol exposure”) OR (“maternal alcohol expos*”)))
#3	(TS= ((”intima media thickness”) OR (”carotid intima media thickness”) OR (”aortic intima media thickness”) OR (”vascular dysfunction”) OR (”atherosclero*”) OR (”vascular stiffness”) OR (”arterial stiffness”) OR (”pulse wave velocity”)))
#4	(TS= ((“children”) OR (“childhood”) OR (“infants”) OR (“adolescents”) OR (“adolescence”) OR (“child”) OR (“neonates”) OR (“neonatal”) OR (“newborns”) OR (“Newborn infants”) OR (“offspring”) OR (“animals”)))
#5	((#1 AND #3 AND #4) OR (#2 AND #3 AND #4) OR (#1 AND #2 AND #3 AND #4))

Table S3: Search strategy developed in PubMed.

Search #	**Search terms**
#1	"maternal smoking" [tiab] OR "maternal smok* OR "intrauterine tobacco smoke expos* [tiab] OR "maternal smoking during pregnancy" [tiab] OR "prenatal smoking" [tiab] OR "prenatal smoke expos*" [tiab] OR "smoke pregnant" [tiab] OR "smoking pregnant" [tiab] "smoking during pregnancy" [tiab] OR "cigarette smoking" [tiab]
#2	"alcohol exposure" [tiab] OR "maternal alcohol exposure" [tiab] OR "maternal "alcohol expos*" OR "maternal alcohol consumption" [tiab] OR "maternal alcohol drinking" OR "fetal alcohol" [tiab] OR "foetal alcohol" [tiab] OR "fetal "alcohol exposure" [tiab] OR "foetal exposure" [tiab] OR "alcohol exposure in utero" [tiab] OR "ethanol" [MH] OR "ethanol exposure" [tiab]
#3	"intima media thickness" [tiab] OR "carotid intima media thickness" [MH] OR "aortic intima media thickness" [tiab] OR "vascular dysfunction" [tiab] OR "atherosclerosis" [MH] OR "atherosclerotic lesions" [tiab] OR "Plaque, Atherosclerotic" [MH] OR "vascular stiffness" [MH] OR "arterial stiffness" [tiab]
#4	"children" [tiab] OR "childhood" [tiab] OR "infants" [tiab] OR "adolescents" [tiab] OR "adolescence" [tiab] OR "child" [MH] OR "pediatrics" [MH] OR "neonates" [tiab] OR "neonatal" [tiab] OR "newborns" [tiab] OR "Newborn infants" [tiab] OR "offspring" [tiab] OR "animals" [MH]
#5	((#1 AND #3 AND #4) OR (#2 AND #3 AND #4) OR (#1 AND #2 AND #3 AND #4))
